# Molecular Dynamics Simulation of Structural Signals of Shear-Band Formation in Zr_46_Cu_46_Al_8_ Metallic Glasses

**DOI:** 10.3390/ma11122564

**Published:** 2018-12-17

**Authors:** Shi-Dong Feng, Keith K. C. Chan, Lei Zhao, Li-Min Wang, Ri-Ping Liu

**Affiliations:** 1 State Key Laboratory of Metastable Materials Science and Technology, Yanshan University, Qinhuangdao 066004, China; limin_wang@ysu.edu.cn (L.-M.W.); riping@ysu.edu.cn (R.-P.L.); 2 Advanced Manufacturing Technology Research Centre, Department of Industrial and Systems Engineering, The Hong Kong Polytechnic University, Hung Hom 999077, Hong Kong; 13901872r@connect.polyu.hk

**Keywords:** metallic glasses, shear band, microstructure, molecular dynamic simulation

## Abstract

The evolution from initiation to formation of a shear band in Zr_46_Cu_46_Al_8_ metallic glasses is presented via molecular dynamics simulation. The increase in number and the decrease in average size of clusters with the quasi-nearest atoms being 0 correspond to the shear-band evolution from initiation to formation. When the shear band is completely formed, the distribution of the bond orientational order *q*_6_ reaches a minimum. The maximum of the number of the polyhedral loss of Cu-centered <0, 0, 12, 0> and the minimum of the number of the polyhedral loss of Zr-centered <0, 2, 8, 5> correspond to the shear-band formation. These findings provide a strong foundation for characterizing the evolution from initiation to formation of shear bands.

## 1. Introduction

The shear band plays a decisive role in controlling the deformation of metallic glasses (MGs) at room temperature, in which large plastic strain is concentrated [1,2,3]. A shear-band process includes formation, propagation, and arrest [4]. Shear transformation zone (STZ)-clusters originate from local atomic regions and intertwine with each other when forming a nascent shear band [5,6]. Following formation, shear bands begin to propagate in two modes [7]. One is synchronous shearing, that is the shear units propagate to the whole shear plane at the same time, and the other is progressive shearing, in which the shear displacement occurs at some random positions and then gradually propagates throughout the sample. Many methods have been tried to suppress shear-band propagation to improve the plasticity of MGs. For example, the self-locking effect of nanocrystals formed during the shearing process can prevent the propagation of shear bands [8]. The deceleration before reaching the maximum shear-band speed provides evidence to suppress the propagation, causing shear-band arrest [4]. Suppressing the shear-band propagation can cause shear-band arrest, which is the reverse process to shear-band initiation. The transient shear-band formation is the first step towards understanding shear bands. Since shear-band formation is very fast, current research is mainly focused on the post-mortem shear bands [9,10].

The structural evolution of shear bands requires nanoscale structural monitoring [4,11]. Molecular dynamics (MD) simulation can reveal the heterogeneous microstructure of MGs at the nanoscale, although the time- and size-scale of MD simulation is usually different to laboratory experiments [12,13,14]. In recent MD simulations, the shear bands can easily form around γ-defects with high shear stress in MGs [15]. Shear-band formation can break specific clusters [5,16]. In addition, the destruction of interconnected icosahedral networks during shear-band formation is revealed by MD simulations [17,18]. The above studies provide for structural changes in shear bands, but specific structural signatures characterizing when and how the shear band is formed are not enough, which increases the difficulty of regulating the shear bands. Due to space and time constraints, the initiation to formation of a shear band is still unknown. Therefore, there is an urgent need to understand the evolution from initiation to formation of shear bands in MGs at the atomic level. In this work, by MD simulation, the effects of cluster networks, bond orientational order, and polyhedral loss on shear-band formation were investigated in Zr_46_Cu_46_Al_8_ MGs.

## 2. Materials and Methods

**Figure 1:**
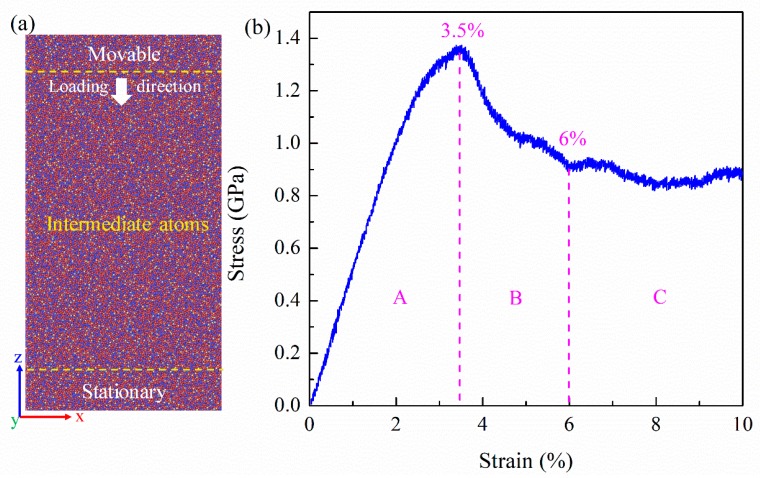
(**a**) The schematic diagram of atomic structure and deformation process. (**b**) The stress-strain curve of the Zr_46_Cu_46_Al_8_ MG divided into three regions, marked as A, B, and C.

Using LAMMPS software (Version: 17 Dec 2016, Sandia National Laboratories, Albuquerque, NM, USA) [19], MD was performed with the reliable empirical embedded atom method (EAM) potential [20]. With reliable empirical potential and excellent glass-forming ability, the composition Zr_46_Cu_46_Al_8_ was adopted [21]. An initial box of 5.8 nm × 5.8 nm × 5.8 nm composed of randomly distributed 4600 Zr atoms, 4600 Cu atoms, and 800 Al atoms was constructed. It was equilibrated at 2000 K and 0 Pa with periodic boundary conditions (PBCs) in three directions within an *NPT* (constant number, constant pressure, and constant temperature) ensemble [22]. The Parrinello-Rahman barostat and Nosé-Hoover thermostat were employed to control the pressure and temperature [23,24], which was quenched to 50 K with a cooling rate of 1.0 × 10^12^ K/s. By replicating 4, 1, and 8 times in *X*, *Y*, and *Z*-directions of the model, a large-sized model containing 320,000 atoms (22.4 nm × 5.6 nm × 44.8 nm) was prepared and followed by annealing at 800 K for 1 ns, and finally quenched to 50 K. The inhomogeneous compression deformation over many cycles was adopted. In each cycle, the model was applied with an instantaneous small-strain and then relaxed for a while. In this work, the rigid atoms at one end along the *Z*-direction of the Zr_46_Cu_46_Al_8_ model were moved 0.018 nm while keeping those at the other end stationary, then the remaining intermediate atoms were relaxed for 10 ps at 50 K, as shown in Figure 1a. This process was executed cyclically until the total strain was 10%, giving a typical strain rate of about 4 × 10^7^ s^−1^. In the deformation process, the *X*-direction was the free surface, and the other two directions were the enforced PBCs. The low temperature of 50 K and the free surface of the *X*-direction facilitated the shear-band formation [5,25].

## 3. Results and Discussion

### 3.1. Stress-Strain Curve

**Figure 2:**
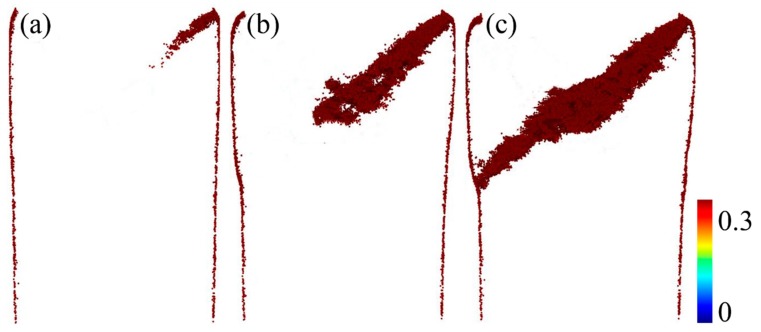
Snapshots capturing the atomic deformation processes at strains of (**a**) 3.5%, (**b**) 5%, and (**c**) 6%. Only atoms with local shear strain *η^Mises^* > 0.3 are displayed.

The stress-strain curve of the Zr_46_Cu_46_Al_8_ MG is divided into three regions, marked as A, B, and C in Figure 1b. Region A is basically composed of linear segments, corresponding to elastic deformation. At the end of region A, the curve deviates from linearity, suggesting the occurrence of irreversible shear transitions. At a strain of 3.5%, the stress reaches a maximum and the subsequent sharp drop corresponds to the shear-band initiation [5]. At a strain of 6%, the stress reaches a steady state and is considered as the strength of a propagating shear band [26,27,28,29]. Therefore, region B corresponds to the shear-band formation. The difference in maximum strength and flow strength indicates the degree of local softening during deformation. Region C corresponds to the shear-band propagation and subsequent stages. Generally, the maximum stress corresponding to the shear-band initiation is obvious, while the stress corresponding to the shear-band formation is usually difficult to determine experimentally. However, in our simulation, there is a saddle point at a strain of 6%, as shown in Figure 1b, which is associated with the shear-band formation. To verify this, the specific process of the shear-band formation is shown in Figure 2.

### 3.2. Atomic Local Shear Strain

Figure 2a–c shows snapshots capturing the atomic deformation processes at strains of (a) 3.5%, (b) 5%, and (c) 6%. Only atoms with local shear strain *η^Mises^* > 0.3 are displayed. The shear band is initiated in the surface at a strain of 3.5%, corresponding to the maximum stress. Further straining induces the formation of half incipient shear bands at a strain of 5%. As the load increases to a strain of 6%, the shear band is completely formed in the MG. Combined with the stress-strain curve in Figure 1, the shear band is initiated at the peak stress, and forms during strain ranges of 3.5% to 6%. The dilation of the structure during the shear-band formation is similar to that in a supercooled liquid state, indicating that the process of shear-band formation is similar to the process of stress-induced glass transition [30]. The saddle point on the stress-strain curve is related to the stress-induced ‘glass transition point’ at a strain of 6%. To explain the phenomenon, the corresponding evolution of the microstructure in the process was studied in detail in the following.

### 3.3. Cluster Analysis

**Figure 3:**
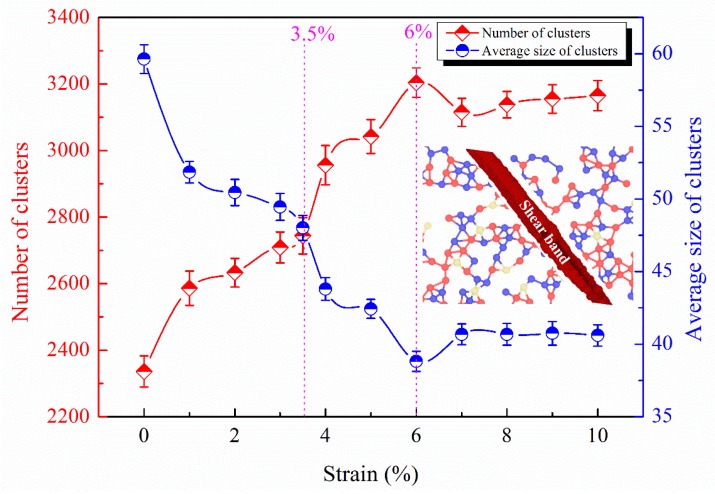
The number and average size of clusters with *N_Q_* = 0 at different strains. The insert shows the schematic diagram of breaking atomic connectivity.

Most polyhedra in MGs exist in the form of links, not in isolation [31]. Therefore, the connection of clusters through the polyhedral nearest neighbors was analyzed. The basic parameters generated by cluster analysis are: number, average size, distribution, and maximum size of clusters. Here, we select atoms with the number of quasi-nearest atoms (QNA) being 0 as the constituent units of the clusters. A pair of QNA should satisfy the following three conditions: (1) They share a common nearest neighbor; (2) their corresponding Voronoi faces of the Voronoi polyhedron centered by their common nearest neighbor share an edge; and (3) they are not the nearest neighbors of each other [32,33,34]. The number of QNAs (*N_Q_*) reflects the disorder degree of the atomic structure [35]. *N_Q_* = 0 suggests that the central atom is subject to many constraints and is not easy to move, belonging to the class of solid atoms. The cluster networks composed of atoms with *N_Q_* = 0 resist deformation [35]. As shown in Figure 3, the number of clusters with *N_Q_* = 0 increases with increasing strain, reaching a maximum at a strain of 6%. The average size of the clusters decreases with increasing strain, reaching a minimum at a strain of 6%. This suggests that the shearing units gradually break the original cluster network composed of atoms belonging to *N_Q_* = 0 as the strain increases, as shown in the insert of Figure 3. Therefore, the average size of the cluster becomes smaller and the number of clusters becomes larger. Since the yield corresponds to the interconnection of the liquid-like cores, a liquid-like layer in the shear plane begins to form at the strain of 3.5%, corresponding to the initiation of a shear band. On the other hand, the breakdown of the icosahedral clusters is also a structural feature of yielding, which proves that the connection and softness of the liquid-like cores provide the origin of the shear band [5]. After yielding, local plastic shearing events, such as STZs, aggregate and link to one another, gradually developing into a mature shear band during the strain range of 3.5% to 6%. When the shear band is completely formed at a strain of 6%, the size and number of the clusters experience minimum and maximum values, suggesting that strains should be easily confined in the shear bands and not easily extended to other undeformed areas. After a strain of 6%, the number of clusters with *N_Q_* = 0 decreases, while the average size of the clusters increase, reconstructing cluster networks composed of atoms with *N_Q_* = 0. Therefore, the stress causes the shear band to change from liquid-like to solid-like, achieving a smooth flow stress state. In addition, after a strain of 6%, the number and size of the clusters remains basically unchanged. During shear-band formation, the elastic energy gradually dissipates in the shear plane, resulting in softening becoming dominant; after which the elastic energy is consumed and the shear band changes from liquid-like to solid-like, resulting in the arrest of the shear band [36,37]. Therefore, our results are consistent with the published results.

### 3.4. Bond Orientational Order

**Figure 4:**
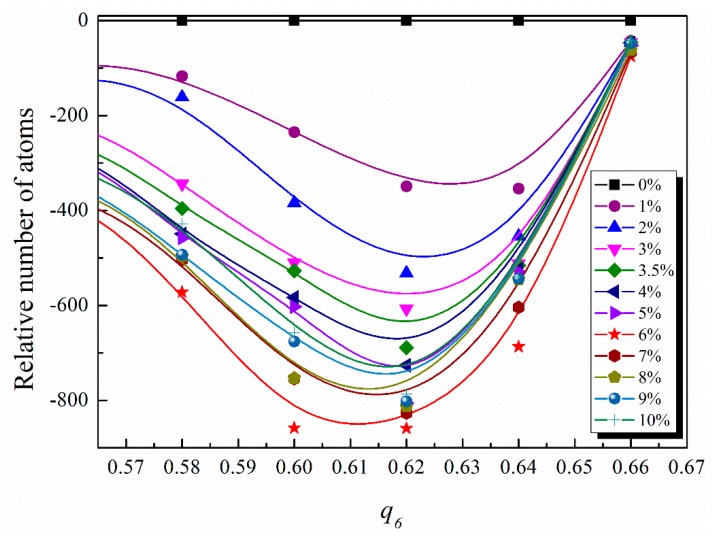
Distribution of relative number of atoms with *N_Q_* = 0 as a function of *q*_6_, which is the number of atoms at different strains subtracted from that at a strain of 0%.

The bond orientational order (BOO) can quantify the orientational symmetry of atomic clusters in disordered systems, and is defined as [38]:

(1) $${\bar{Y}}_{lm}=\frac{1}{N_{i}}\overset{N_{i}}{\underset{j=1}{\sum }}Y_{lm}\left(\theta \left(r_{ij}\right),\;\emptyset \left(r_{ij}\right)\right)$$

(2) $$q_{l}=\sqrt{\frac{4\pi }{2l+1}\sum_{m=-l}^{l}{\bar{Y}}_{lm}{\bar{Y}}_{lm}^{*}}$$

where, *Y_lm_* are the spherical harmonics, *N_i_* is the number of nearest neighbors of atom *i*, the angles *θ* and *ϕ* are the standard spherical polar angles, and ***r****_ij_* is the position vector between atom *i* and *j*. The value *q*_6_ = 0.663 represents a perfect icosahedron [39,40]. The Cu-centered icosahedra in the shear band are severely destroyed and some other polyhedra are formed, whose first nearest neighbors are characterized by *q*_6_ < 0.57 [41]. Kim and Ryu found that Cu atoms with higher *q*_6_ have higher structural rigidity and are more resistant to deformation [42]. In our work, the selection range of *q*_6_ is set from 0.57 to 0.663 to characterize the resistance to deformation. The relative number of atoms with *N_Q_* = 0 as a function of *q*_6_ between 0.57 and 0.663 is shown in Figure 4, which represents the number of atoms with *N_Q_* = 0 at different strains subtracted from that at a strain of 0%. As the strain increases, the relative number of atoms gradually decreases. At a strain of 6%, the relative number of atoms reaches the minimum. After a strain of 6%, the relative number of atoms gradually increases with increasing strain. As mentioned above, atoms in the region of high *q*_6_ (0.57–0.663) possess the properties of high rigidity and low-participation deformation. At a strain of 6%, the *q*_6_ reaches a minimum, indicating the weakest resistance to deformation and the largest fraction of the atoms participating in the deformation. This corresponds exactly to the shear band being completely formed and needing to propagate.

### 3.5. Polyhedral Loss

**Figure 5:**
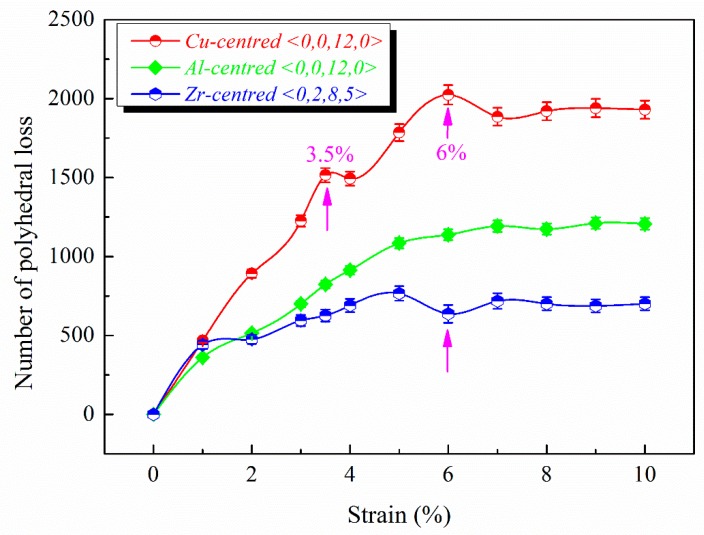
The number of polyhedral loss of Zr-centered <0, 2, 8, 5>, Cu-centered <0, 0, 12, 0>, and Al-centered <0, 0, 12, 0> at different strains.

The structural evolution of the shear bands is characterized from the number, size, and symmetry of the clusters, and the specific structural changes of clusters are characterized in more detail in the following. Voronoi polyhedra (VP) are expressed using the indexes <*n*_3_, *n*_4_, *n*_5_, *n*_6_>, in which *n_i_* represents the number of *i*-edged faces [43]. The <0, 0, 12, 0> VP correspond to full icosahedra in MGs and are closely related to the deformation [16,44]. Compared to any other short-range order, the high content of icosahedra corresponds to a high yield strength and an enhanced shear resistance [7,14]. The <0, 2, 8, 5> VP tend to be connected to each other and form interpenetrating network structures in the shear band in Cu_64_Zr_36_ MGs [25]. Therefore, the <0, 0, 12, 0> and <0, 2, 8, 5> VP are selected to represent polyhedral loss. The polyhedral loss refers to the loss of the polyhedral number of the nearest neighbors of each atom at different strains compared to that at a strain of 0%, which reflects the damage degree of the cluster networks. As shown in Figure 5, it is found that there is an extreme point and a maximum of the number of the polyhedral loss of Cu-centered <0, 0, 12, 0> at the strain of 3.5% and 6%, respectively. During plastic deformation, the icosahedral fraction in the shear bands decreases [5,45]. The extreme point for the number of polyhedral loss of Cu-centered <0, 0, 12, 0> at a strain of 3.5% corresponds to the beginning of shear-band formation; and the maximum at a strain of 6% suggests that the shear band is completely formed, leading to the icosahedral damage reaching the maximum. This is because, as the strain increases, new STZs continue to be produced, which sacrifice the icosahedra. There is a minimum of the number of polyhedral loss of Zr-centered <0, 2, 8, 5> at a strain of 6%. Our preliminary results indicate that Zr-centered <0, 2, 8, 5> VP tend to be connected with each other, forming an interpenetrating backbone in the shear band. The minimum for the number of polyhedral loss of Zr-centered <0, 2, 8, 5> at a strain of 6% suggests that the shear band has been completely formed, and the newly formed Zr-centered <0, 2, 8, 5> clusters cancel out the clusters destroyed by the previous deformation. As the strain increases, the number of polyhedral loss of Al-centered <0, 0, 12, 0> gradually increases. However, the Al-centered <0, 0, 12, 0> VP remain basically unchanged after a strain of 5%, and there is no extreme point for the number of polyhedral loss of Al-centered <0, 0, 12, 0>. This is because the metallic bonds between Cu and Zr are more flexible and easier to break and reform than the covalent-like bonds of Al-X [46,47,48]. After a strain of 6%, the number of polyhedral loss of Zr-centered <0, 2, 8, 5>, Cu-centered <0, 0, 12, 0>, and Al-centered <0, 0, 12, 0> VP all reach stable values, as shown in Figure 5, which suggests that the solid-like structure is reconstructed, and the MG matrix resists the shear band sliding. As seen in Figure 3, the number of clusters with *N_Q_* = 0 decreases after a strain of 6%, while the average size of clusters increases, reconstructing cluster networks composed of atoms with *N_Q_* = 0.

## 4. Conclusions

The evolution from initiation to formation of a shear band in Zr_46_Cu_46_Al_8_ MGs was investigated through a molecular dynamics simulation. The shear band is initiated at the peak stress, and forms during the strain range of 3.5% to 6%, characterized by atomic local shear strains *η^Mises^* greater than 0.3. The maximum number and the minimum average size of clusters with *N_Q_* = 0 at the strain of 6% corresponds to the shear-band formation. Further, the distribution of *q*_6_ reaches a minimum, indicating the weakest resistance to deformation and the shear-band formation. The maximum of the number of the polyhedral loss of Cu-centered <0, 0, 12, 0> and the minimum of the number of the polyhedral loss of Zr-centered <0, 2, 8, 5> at a strain of 6% suggest that the icosahedral damage reaches the maximum and the newly formed Zr-centered <0, 2, 8, 5> clusters cancel out the clusters destroyed by the previous deformation, corresponding to the shear-band formation. The specific structural signatures characterizing when and how the shear band is formed provide a more in-depth understanding of the formation mechanism of shear bands.
